# In Search of an Audience: Popular Pharmacies and the Limits of Literate Medicine in Late Seventeenth- and Early Eighteenth-Century Russia

**DOI:** 10.1353/bmh.2015.0099

**Published:** 2015

**Authors:** Clare Griffin

**Keywords:** Russia, early modern, medical books, literacy

## Abstract

This article addresses the question of the limits of literate medicine in Europe, through an examination of the Russian literate medical world of the late seventeenth and early eighteenth centuries. Russian courtly medicine had been dominated by Western Europeans from the 1480s, but in the early eighteenth century new licensing arrangements solidified the presence of these foreigners in the wider Russian medical world. Foreign medical practitioners took advantage of this development, aiming works at an increasingly large proportion of Russian literate society. These works, along with satirical and religious works emulating or deriding medical texts, show how by the 1720s the limits of literate medicine in Russia lay not at the edges of official court medicine, but rather at the edges of literate society.

In early modern Europe a notable section of health care information was encoded in and transmitted by texts, texts that increasingly were not just for trained practitioners but also for use by sufferers themselves. Yet the majority of laypersons, and indeed a fair proportion of healers, in all European countries could not read, and treated themselves and others[Other P-705] using methods and recipes conveyed in the oral tradition. There were thus two medical worlds, literate and nonliterate, which coexisted, overlapped, but also came into conflict. Nowhere was the existence of these two worlds more striking than in Russia. Literate medicine was Western European, practiced by foreigners, and dominated by the court’s medical department, which provided services to the court and the army; nonliterate healing, upon which the vast majority of Russians relied, was practiced by native Russians, and was unofficial and often illicit.^1^ This situation was partly a product of contemporary Russian views on books and reading. Reading itself did not have the same status as in the humanist West: in Kievan Rus’ reading had been seen not as an infallible path to God, but as a double-edged sword that was as likely to mislead as to enlighten.^2^ Even during the seventeenth century, when books and book culture became a more important part of Russian life, the possession of writings was sufficiently suspicious to cause numerous accusations of witchcraft based only on the ownership of texts.^3^ For Muscovites, reading and writing were manual skills and not cultural assets: the court possessed clerks who could perform these skills for nobles, and “illiteracy” was no barrier to state service by a noble. When reports were presented to the tsar and his noble council, they were commonly spoken to the council, rather than being read by each individual.^4^ This situation began to change across the seventeenth century, and in particular into the eighteenth century under Peter the Great, who supported the adoption of various Western European cultural artifacts, including the concept of literacy as a fundamental part of being a nobleman. Nevertheless, literacy only slowly became an essential part of life for the Russian elite: Gary Marker has estimated that literacy may have been as low as 3 to 5 percent of the population even by the late seventeenth century; Christoph Witzenrath puts it rather higher, between[Other P-706] 11 and 13 percent.^5^ Of the nine nobles Peter the Great appointed to his newly created Senate in 1711, one was unable to sign his own name, yet such a lack of literacy was apparently no bar to high state service.^6^ Despite his reliance upon nonliterate advisers, Peter saw literacy and education as vital to his project of reshaping Russia. Peter’s time as sole ruler of Russia (1696–1725) was thus a liminal period for literacy in Russia, a period of transition from the old model of literacy as marginal and suspicious, to the Western model of reading as central and desirable.

During this liminal period, pharmacy texts—medical books instructing the reader on how to create complex medicines using distillation and similar technical processes—appeared in Russia (and in Russian), which were addressed to various sections of Russian society. All the works are manuscripts, following the general trend for Russian works of this period: printing was state-controlled and, even into the eighteenth century, was primarily used for producing standardized, hard to fake documents; the first medical work was printed in Russia only in 1732.^7^ These little-studied manuscripts were based on Western models, and designed not for medical professionals, but for laypersons seeking to treat themselves or others.^8^ The prefaces in these works, often only brief sections of text that were common in popular medical works across early modern Europe, are both direct and specific in their identification of their audience, including, most intriguingly, service persons and ordinary Russians, people who were rarely functionally literate. These prefaces are the focus of this article because they give us a rare and valuable insight into the audience of popular medical books, as that audience was imagined by the authors of said books. What, then, can these texts tell us about the[Other P-707] limits of literate medicine in Russia? The prefaces give us the history of an assumption—the assumption, made by the compilers of these texts, that there were literate Russians without medical training who could and would acquire, keep, read, and use Western European medical texts. The groups addressed by these texts show us that medical writers assumed the limits of literate medicine not to be the court, but to extend far beyond it, to encompass effectively all literate Russians.

## Medicine and Book Culture in Early Modern Russia

Literate medical practitioners in early eighteenth century Russia were a minority: there were folk practitioners of various stripes, and also Christian healers, who often used religious texts in their healing practices, but the only representatives of university-educated, literate medicine were among the foreign (Western European) medical practitioners brought to Russia by members of the court elite. Indeed, far from all medical practitioners at the Russian court had a university education; many others, in particular the surgeons and apothecaries, had guild training, and some apparently had no formal training at all. These men—university-educated and otherwise—came to Russia in increasing numbers from the 1480s on. By the 1580s their work had been formalized into a court medical department, known in the seventeenth century as the Apothecary Chancery (*aptekarskii prikaz*), which provided a range of services to the tsar and his family, courtiers, and other servitors, primarily soldiers.^9^ From 1654 the Apothecary Chancery began training Russians in the preparation of medicines and also surgery, but this was an apparently limited program[Other P-708] that did not lead to a new, native caste of formally trained Russian medical professionals; even after 1654, literate medicine was long dominated by Western Europeans.^10^ These Westerners, and the Apothecary Chancery where they worked, played a large role in shaping Russian medicine. Up until the end of the seventeenth century, the Apothecary Chancery was Russia’s sole official medical institution, healing both courtly and military patients and providing the court with reports on medical matters, such as plague. From 1673, the department also sold medicines to Muscovites through its own pharmacy. When Peter the Great began his bureaucratic revolution in the late 1690s, medicine was among the areas transformed: during the seventeenth century, the department had always been run by a high-ranking noble; from 1696 Peter left the department in the hands of an administrator. Moreover, the virtual monopoly of the Apothecary Chancery was undermined by dividing up its duties among new institutions. Military medicine was provided by the army and navy’s own medical services (from 1716 and 1720, respectively), court medicine was provided first by the Medical Chancellery (from 1714) and later the Medical College (from 1725), and the preparation of reports was assigned largely to the Academy of Sciences (founded in 1724).^11^ Moreover, he also licensed private practitioners—apothecaries—for the first time in 1701.^12^ The latter development is particularly significant, as it marks the start of more intensive involvement of Western European, literate medicine in the Russian medical world. It was during this period of reshaping Russian literate medicine that these popular pharmacies were composed.

The Apothecary Chancery’s literate medical practitioners necessarily relied upon texts for their medical expertise. Medical texts were imported into Russia from Western Europe, and a number were translated into Russian, in particular for the use of the Russian students of the Apothecary Chancery. Examination of medical texts in Russia is at a fairly early stage: an important variant of the most famous early modern Russian-language medical text, the herbal known as the Garden of Health (*Blagoprokhladnyi Vertograd*), was identified only in 2000.^13^ Although V. F. Zmeev put together a catalogue of early Russian medical texts in the 1890s, he himself was[Other P-709] aware that his list was incomplete, and no one has yet significantly revised his work.^14^ Various important medical works were owned by the Apothecary Chancery and its successor the Medical Chancellery: Galen’s work on medical theory and Vesalius’s famous anatomy textbook were present in the Apothecary Chancery library, as were a range of Western European pharmacy texts.^15^ Vesalius’s anatomy and Braunschweig’s key work on distillation were actually translated into Russian, although the extent of their circulation is unclear.^16^ In addition, the earliest version of a Russian-language pharmacy text known only as the *Pharmacopoeia* was apparently compiled for the use of Apothecary Chancery students and staff (see below). Significantly, although these medical works available in Russia before the 1690s could have been used by an amateur enthusiast, none of them were specifically aimed at laypersons seeking to heal themselves.

The texts we focus on here appeared during Peter’s bureaucratic revolution in Russian medicine, from the 1690s through to the 1720s, and are all aimed at laypersons seeking to heal themselves. Interestingly, these works were composed by staff from the Apothecary Chancery, the very institution that was being superseded by Peter’s reforms. Daniel Gurchin was the most prolific of such compilers, creating or co-creating at least seven such texts. Relatively little is known about Gurchin. He was apparently Polish—his original surname being Hurczyn—as were many apothecaries and surgeons working at the Russian court, but almost nothing is known of his life before he came to Russia. He certainly worked in the Apothecary Chancery for some time in the 1690s and 1700s, and in 1701 he was the second man to open a private apothecary shop under Peter’s new legislation.^17^ Gurchin created or compiled pharmacy texts throughout his career in Russia. T. V. Panich has demonstrated that Gurchin compiled[Other P-710] the Russian *Pharmacopoeia* in 1676, and helped Afanasii of Kholmogory to create the *Extract from Doctors’ Knowledge* (*Reestr iz dokturskikh nauk*) in 1696.^18^ Gurchin continued his collaborative work with Laurentius Blumentrost the elder, with whom he produced two versions of the *Domestic and Field Pharmacy* (*Domovaia i pokhodnaia apteka*), one for royal consumption (in 1696) and the other for more humble readers (extant in copies from the 1720s on). Gurchin’s productions of the eighteenth century were heavily focused on a humble readership: he revised the *Pharmacopoeia* for a lay audience (copies date from the early eighteenth century on); in 1708 he was the sole compiler of the *Pharmacy for Transport or Service* (*Apteka obozovaia ili sluzhivaia*);^19^ he is also the probable compiler of the *Small Domestic Pharmacy* (*Aptechka domovaia*; early eighteenth century) and the *Large Domestic Pharmacy* (*Opteka domovaia bolshaia*; 1705). Gurchin’s authorship of the later works is less firmly established than for the earlier works: in the case of the *Pharmacopoeia* and *Extract from Doctors’ Knowledge*, there is evidence from both the texts themselves and a series of letters between Gurchin and Afanasii of Kholmogory; in the case of the *Domestic and Field Pharmacy, Pharmacy for Transport or Service*, and *Small Domestic Pharmacy* Gurchin is named as the compiler in the texts themselves; in the case of the *Large Domestic Pharmacy*, Gurchin’s authorship is solely based on its textual similarity to his earlier works.^20^ Even allowing for the issues in conclusively attributing some of these works to Gurchin, the current level of knowledge about Gurchin and these texts supports his identification as a significant producer of Russian-language pharmacy texts. He may also be the same Daniel Gurchin who wrote a poem in honor of Peter the Great, which fact would further support the identification of Gurchin as a notable medico-literary figure of Petrine Russia.^21^

One of Gurchin’s co-compilers, Laurentius Blumentrost the elder, as he called himself, was similarly a foreign medical practitioner working in the Apothecary Chancery; his son, Laurentius Blumentrost the younger (b. 1655), followed in his father’s footsteps, working in the Apothecary Chancery from 1685.^22^ Yet the Blumentrosts, as physicians and not[Other P-711] apothecaries, were less well placed to take advantage of Peter’s reforms. Moreover, by the start of the period under consideration Blumentrost the elder was already in his seventies (he would die in 1705, at the age of eighty-six). The primary evidence of Blumentrost the elder’s involvement in the production of these Russian-language pharmacy texts is that the *Domestic and Field Pharmacy* was based on a work Blumentrost had written and published in Latin in 1667: the *Pharmacotheca domestica et portatilis*, published in German as the *Haus- and Reis-Apotheken* in 1715.^23^ It is significant that none of the lists of medical works owned by the Apothecary Chancery list Blumentrost’s work, and so Blumentrost himself is the logical source of the original from which the Russian version was made. As an old man with an established reputation at court, Blumentrost’s involvement in these texts is likely to have been that of the senior partner, providing content and lending his name to the texts, but leaving much of the actual work to Gurchin. The presence of Blumentrost’s son in the Apothecary Chancery may have in part led to the elder Blumentrost’s decision to be involved in this project at such an advanced age, perhaps with the thought of bequeathing to his son a reputation for medical authority both outside and within the court. The work of Blumentrost and Gurchin in creating and promoting Russian-language medical texts, although problematic to pin down exactly, is hugely important in revealing aspects of literate medicine in Russia that would otherwise be lost.

## Appropriate Literacy: Texts for Patrons

The earliest of Gurchin and Blumentrost’s texts were aimed at patrons among the noble and royal elite. The targeting of an elite audience fits into both the Muscovite gift economy in which texts were a significant currency, and the circulation of knowledge in Russia. Gifting books, and medical knowledge, was a common part of client-patron relations across Europe in this period.^24^ Gifting of books in general grew in importance in late Muscovy, as literacy increasingly became seen as a desirable attribute for a noble (*boyar*); tsars led the way, with books being gifted to tsars and their heirs on a variety of topics. Interestingly, despite the overwhelming proportion of Russian books of this period that were mainly or exclusively religious in focus, such works were often secular or practical in nature,[Other P-712] with the future tsar Alexei Mikhailovich being gifted a textbook on geometry as a child.^25^ Medical books fit into the same broad scheme of practical works. Significantly, such practical works were commonly based on or translations or adaptations of Western European works. Russia had long been somewhat apart from Latinate Western Europe, divided by religion and language, but from the 1480s on the Russian court made significant efforts to engage with their Western neighbors. These court contacts gradually developed into changes in Russian culture: by the mid- to late seventeenth century, such Western contacts began to produce Westernization in certain areas of Russian life, which became both more extensive and intensive in the eighteenth century. Western books and expertise played a key role in that process. The compilers of these Russian-language Western-style medical texts evidently believed that a medical text would be an appropriate gift for their patron, revealing that the Muscovite elite were believed by such compilers to have some interest in literate medicine, either as self-help, as intellectual endeavor, or simply as a piece of conspicuous consumption, as a badge of membership of the increasingly literate and Westernized Russian elite of the late seventeenth and early eighteenth centuries.

These texts also followed established norms of knowledge circulation. The Apothecary Chancery, like other Russian court departments, commonly wrote reports on a range of subjects for use within the department, and also within the court or chancery system. The chancery system, the collection of all Russian court departments and regional administrators, was an early bastion of literacy in Muscovy.^26^ Although chancery reports were often read to high-ranking councilors, information, orders, and reports were exchanged by means of the written word. These reports were meant to be consumed by department heads, who were almost exclusively noble.^27^ Some reports had even more exalted readers, being sent to the tsar himself, for example a series of reports on the purchase of unicorn horn.^28^ In targeting an elite audience, Gurchin and Blumentrost were tapping into established forms of patronage through literate exchange.[Other P-713]

In the 1690s, Gurchin and Blumentrost directed their attentions to the very top of the Russian hierarchy, dedicating a text to Peter the Great. This work was presented to Peter in 1698, and later to his son, Tsarevich Aleksei, and survives in two early eighteenth-century copies.^29^ The royal *Domestic and Field Pharmacy*, as the text is known, brings together several medical works, introduced by a lengthy preface. Despite the importance of the recipient of this medical text, there is no published edition, and little scholarly attention has thus far been devoted to it.^30^ This lacuna is all the more notable, as Peter was known for having a personal interest in Western medicine, alongside his interests in other areas of European knowledge: Peter studied anatomy, and purchased the anatomical collection of the Dutch physician Frederik Ruysch, which formed the basis for Peter’s Kunstkamera, a museum of natural oddities that was open to the public.^31^

Despite the obvious interest that such a work should hold for a lover of practical Western European knowledge such as Peter, the text goes to some length to justify itself as a text fit for a tsar. Both manuscripts of the royal *Domestic and Field Pharmacy* begins with an extensive preface that explains why medicine should be of interest to the reader.^32^ The text calls on several ideas to legitimize itself. First, it invokes religion, stating, “By God’s will food and wealth is given by the earth, especially in certain realms … but above all [the ability to] retain human health.”^33^ It further relies upon royal legitimacy, stating that both European monarchs such as Rudolph II, Holy Roman Emperor (1576–1612), and biblical kings like Solomon, had an interest in medicine: “[I] humbly remind [you of the validity of alchemy], if you allow [me], to discuss [such matters using] Biblical parables in the Book of Moses … how Moses made powder from unburnished gold and gave to people to drink in water, and how Tsar Solomon held this knowledge of ores and all herbs and their actions[Other P-714] in great honor [as] written in the Book of Solomon.”^34^ Thus the reader is enjoined to value medicine, and in particular this book of medicine, because of relevant precedence.

The precedents the prefaces choose to cite are revealing of the compilers’ assumptions regarding their audience. Medicine is here presented as both godly and imperial, appealing to two serious concerns of the Christian ruler. Medicine, in Russia as elsewhere, had a difficult relationship to the Church and to religion. Some texts, including the royal *Domestic and Field Pharmacy*, promoted medicine as God’s remedies left on earth for the benefit of mankind; others, such as the popular Russian household text the *Domostroi* (discussed below), denigrated it as obviating God’s trials through which believers were to achieve the Kingdom of Heaven. This preface, in so pointedly arguing the former, implicitly acknowledges the existence of the latter view. The question of the appropriateness of the study of medicine to the ruler is somewhat different. Here the issue is about who should read medical texts: it was more common for a ruler to employ medical practitioners than to be learned in medicine himself. In arguing for a medical text as appropriate reading matter for a ruler, the text acknowledges that there is a contemporary view that medicine is to be practiced by artisans for the benefit of the rich and powerful, but not to be learned by them. The text, arguing for medicine to be seen as fit and Godly reading material for a tsar, views Peter as religious, and concerned over the proper limits of behavior for a monarch.

The idea of appropriate knowledge, so important to the royal *Domestic and Field Pharmacy*, also appears in another work aimed at a patron, the *Extract from Doctors’ Knowledge*,^35^ first compiled in 1696 by Archbishop Afanasii of Kholmogory and Daniel Gurchin.^36^ The *Extract from Doctors*’[Other P-715] *Knowledge* was dedicated to Fedor Matveevich Apraksin, then military governor (*voevoda*) of Dvina. Afanasii’s Archbishopric, Kholmogory, was located near Dvina, and so Afanasii likely dedicated the *Extract from Doctors’ Knowledge* to Apraksin due to their links as provincial leaders. Thus, unlike the *Domestic and Field Pharmacy* presented to Peter as a gift from underlings to their master, the *Extract from Doctors’ Knowledge* was a gift between equals.

Despite the difference of function between the *Domestic and Field Pharmacy* and the *Extract from Doctors’ Knowledge*, the latter also relies on the concept of appropriate knowledge. The preface states that it contains knowledge about “which medicines should be owned to [combat] human weaknesses and for what purpose those medicines are, and how to make vodkas against human weaknesses and from which herbs, and so [how to make] medicines and from which things, and what power they have, as is appropriate for Your Excellency the Count and Close Steward Fedor Matveevich Apraksin to have.”^37^

The *Extract from Doctors’ Knowledge* does not justify itself in as much length as the royal text, as its preface consists of only a paragraph rather than the several pages to which the preface to Peter’s work extends, but nevertheless it relies on the same idea: that medicine as an appropriate type of knowledge to be gifted must be defended. These two texts demonstrate that the compilers of these texts believed that medicine was an appropriate form of knowledge, but feared that the audiences they had chosen would not share their enthusiasm.

The issue of appropriate knowledge takes us back to the problems of literacy in Russia. Possession of books and letters, especially in a foreign language, were one of the reasons a person could be charged with witchcraft. This applied not only to peasants and other lowly Muscovites, but even high-ranking courtiers: A. S. Matveev, former adviser to the tsar, was accused of magic on such a basis in 1676; Jacob Bruce, one of Peter’s closest confidents, was commonly associated with book magic.^38^ Matveev and Bruce’s cases were examples of the Russian fear of *chernoknizhestvo*, black book magic, a phenomenon which reflects the problems of literacy[Other P-716] in Russia.^39^ Valerie Kivelson interprets this phenomenon as linked to the issues of hierarchy and control: “The horror of *chernoknizhestvo* displayed in intensified form a far more generalised fear of literacy run amok, of illicit copying and circulation of texts, and of the capacity of writing to serve the disruptive ends of subversive individuals instead of the authorized goals of sanctioned hierarchy.”^40^ Blumentrost, Gurchin, and Afanasii of Kholmogory’s texts, as foreign works on natural objects, would thus seem dangerously close to *chernoknizhestvo*. However, no witchcraft trial lists these specific texts, or any others linked to the Apothecary Chancery. In part, this might be due to the role of foreign physicians at court: recruitment documents constantly stress the need for “knowledgeable people,” and a key duty of Apothecary Chancery medical practitioners was the production of reports for use in the department and elsewhere, including in advising the tsar. The place of foreign physicians in the Muscovite hierarchy was fundamentally linked to the provision of knowledge, and so texts produced by them were unlikely to have been considered suspect. However, there was still a danger of such an association being made. Hence the repeated emphasis on appropriate knowledge, an affirmation that these texts were legitimate and sanctioned, not illicit and subversive.

The idea of appropriate knowledge is further developed in a later section of the royal *Domestic and Field Pharmacy*. Peter’s text mostly consists of pharmacy sections: the *Domestic and Field Pharmacy* itself, and the *Book of Preparing Medicines and Vodkas* (*Kniga glagolemaia lekarstv stroeniiu i vodam*).^41^ Following these two pharmacy sections is a much more unusual type of medical text for Russia, the *Brief Description of Thirty Rules for Health* (*Kratkoe opisanie tridesiat’ pravil k zdraviu*).^42^ As the title suggests, this text consists of thirty aphorisms on how best to preserve one’s health, including rules detailing from which persons it is appropriate to take medical advice: “Do not listen to any unskilled neighbour or kinsman for advice on medicines….^43^ Do not allow yourself to be healed by young healers or old women, and if an illness or injury falls upon you, always seek the advice [of those] skilled in medical matters.”^44^[Other P-717]

Irregular healers were of constant concern to European physicians as competitors, a concern that also existed in Russia: a number of unlicensed practitioners were prosecuted by the Apothecary Chancery in the 1670s, 1680s, and 1690s.^45^ Such practitioners were also a problem in the recruitment of Apothecary Chancery staff, with physicians vociferously—although not always successfully—protesting the employment of individuals whom it saw as unsuitable, such as the 1685 investigation into Ivan Drescher.^46^ The aphorisms concerning appropriate medical advice in the *Brief Description of Thirty Rules for Health* thus seem to have reflected a genuine concern on the part of Apothecary Chancery medical staff over irregular practice. It should also be noted that Peter did take action against some irregular medical practice, eventually establishing private apothecary shops with official licenses in 1701, only a few years after receiving this text.^47^ Whether the physicians’ complaints were the cause of such developments or not, inclusion of this text in the royal *Domestic and Field Pharmacy* undoubtedly demonstrates an urge to shape Peter’s views on appropriate medical practice.

The aphorisms on medical practitioners contained in the *Brief Description of Thirty Rules for Health* do not only focus on whom not to consult—“young healers or old women”—but on whom one should consult—“[those] skilled in medical matters”—meaning Western-trained physicians and apothecaries such as Gurchin and Blumentrost. These men had good reason to promote themselves and their practices in the late 1690s. Prior to Peter’s reign, tsars had appointed high-ranking courtiers and close relatives to the head of the Apothecary Chancery; in stark contrast, from 1696 Peter left the department in the control of an administrator.^48^ Such a situation was far from inevitable: several members of Peter’s All-Drunken Council, seen by Ernest Zitser as Peter’s inner circle, also held positions in the chancery system and its replacement, the councils.^49^ The[Other P-718] appointment of an administrator to the directorship of the Apothecary Chancery does thus seem to have reflected Peter’s disinterest in this particular institution, a disinterest that eventually led to the abolishment of the department. The compilers of this text, Blumentrost and Gurchin, as Apothecary Chancery employees, may have been concerned that the Apothecary Chancery would not play such an important role at court under Peter as it had done previously. Indeed, such concerns might help to explain the involvement of the well-established Blumentrost the elder in this project. Having worked at the Russian court for many decades, Blumentrost lived to see the importance of the Apothecary Chancery diminish, perhaps threatening his son’s future livelihood. This change in the administration of the department might then help to explain why Blumentrost chose to return to the creation of medical books so late in life. The *Brief Description of Thirty Rules for Health* was thus included in the work presented to Peter for two reasons: to warn against irregular practitioners, and to promote Russia’s existing official medical institution, the Apothecary Chancery.

Works presented to patrons in the 1690s thus reveal a deep-seated concern of their compilers concerning the views of their intended audience on medicine. Gurchin and his colleagues took pains to reassure their readers both of the appropriateness of medical knowledge to their religion and their social status, and of its utility. Previously, the Russian elite had primarily encountered literate medicine through the medium of the Apothecary Chancery’s medical practitioners. These texts aimed to bring them into closer contact with literate medicine, through direct access to texts. These texts then do not show an expansion of the sphere of literate medicine, but rather the intensification of elite contact with literate medicine.

## Practical Literacy: Works for Soldiers

Gurchin and Blumentrost also compiled medical texts for soldiers. This group, like the noble patrons discussed above, had access to literate medicine prior to the composition of these texts. In this context “soldier” was unlikely to mean the rank-and-file foot soldiers of the army: most of the Russian army was composed of men from humble backgrounds, who would not have been able to read. Their commanders were mostly Russian nobles (although there was an influx of foreign mercenaries under Peter) who were, as a group, more literate than their men. Commanders, as noblemen, were also more able to afford manuscripts and medicines than ordinary soldiers, as both manuscripts and medicines were relatively[Other P-719] expensive commodities in the early eighteenth century. Thus the audience of these “soldiers’” texts was not too far removed from the noble patrons discussed above.

Like the noble patrons, soldiers had previously had access to literate medicine, through the provisions the Apothecary Chancery made for the army. These texts both acknowledge that system, while revealing a problem with it. Gurchin’s early eighteenth-century *Pharmacy for Transport or Service*, aimed at military servitors and baggage train staff, notes that it was a text “with which in the absence of a surgeon [they] might help themselves during any of their own or their horse’s infirmities.”^50^ As the text is aimed at servitors, it would seem strange that there is an assumption that medical help would not be available, as the Russian army did indeed have military surgeons. However, there were constant problems with the quality and quantity of such surgeons, and this statement reflects such issues. The establishment of the school for field surgeons in 1654 was meant to increase the numbers of regiments who had a surgeon with them, but the limited information available suggests that the Apothecary Chancery’s supply of these men still lagged behind demand in the decades up until the reorganization of Russian military medicine in the 1710s and 1720s.^51^ The small numbers of field surgeons may have had a detrimental effect on the Russian army: John T. Alexander has proposed that the failure of Prince V. V. Golitsyn’s campaigns in the Crimea in 1687–89, and of Peter’s siege of Azov in 1695, can both be linked to inadequate medical provision.^52^ Thus although Russian military servitors in theory had access to literate medicine through army surgeons, in reality that access was limited and problematic. Medical texts aimed at commanders were likely an attempt to compensate for the problems inherent to Russian military medicine in the late seventeenth and early eighteenth centuries, by providing the officers with the basic knowledge necessary to treat themselves.

The utility of a medical text to a Russian soldier is then clear, but why would a medical practitioner choose to target soldiers as an audience for their text? It should be remembered that both Gurchin and Blumentrost were employed by the court, and so these works could have been commissioned to fulfill government aims. Conversely, there is significant evidence that these men could have been motivated by commercial concerns. As noted above, official practitioners like Gurchin and Blumentrost felt[Other P-720] under threat by unofficial healers, who could flourish in the absence of official medical practitioners. Medical texts could help to maintain support for their medical practice even if the practitioners themselves were not directly available to the patient. Gurchin, from 1701 a licensed private apothecary, in particular had commercial reasons for promoting Western medical practice in the absence of Western medical practitioners. It thus seems likely that Gurchin and Blumentrost’s texts fulfilled two complementary aims: the government project of providing medical expertise to the army, and the personal, commercial project of promoting their private medical practices.

As well as a fundamental understanding that soldiers required medical knowledge to survive their campaigns, these works betray further conceptions the compilers had about their audience. Gurchin’s *Pharmacy for Transport or Service* notes that it was “compiled in a concise fashion.”^53^ Military men spent much of their time traveling, and so a “concise” work would be desirable for its portability. Indeed, one copy of the *Pharmacy for Transport or Service* appears to have been specially designed as a “travel” book: the Russian National Library manuscript is only half as wide as a standard quarto, and was originally slightly taller (it was cropped, apparently when later bound into the eighteenth-century miscellany where it is currently found), presumably to make it more portable.^54^ Practical considerations are also acknowledged in the preface’s statement that the *Pharmacy for Transport or Service* is a text “with which in the absence of a surgeon [they] might help themselves during any of their own or their horse’s infirmities.”^55^ This statement is, in effect, a justification of the text, as it highlights why a serviceperson would wish to own or use such a work. Unlike the much more extensive justification of medicine in Peter’s royal text, the preface to the *Pharmacy for Transport or Service* is not concerned with issues of religion or appropriate knowledge in justifying medicine, but simply makes a practical claim: this text is helpful. Gurchin thus views his service person audience as having mainly practical concerns in mind when considering healing.

The *Domestic and Field Pharmacy* was similarly constructed according to the specific needs of its audience.^56^ This work existed in two forms: the[Other P-721] royal version for Peter the Great and his son Tsarevich Aleksei discussed above, and a standard version intended for servitors. Although both originate from Blumentrost’s original Latin work, it is probable that the translation and adaptation of these works were undertaken by Gurchin. There are notable differences between the royal and standard versions of this text: the royal versions prepared for Tsar Peter and Tsarevich Aleksei have one long section, whereas the standard version of the *Domestic and Field Pharmacy* is split into two sections. Moreover, the royal version features recipes that call for high-status and expensive ingredients such as gold; these recipes are not found in the standard version.^57^ The creation of multiple versions of one text according to audience was common practice in Europe. In 1605 Francis Bacon wrote the *Advancement of Learning*, a text advocating the greater use and official regulation of natural philosophy, which was both enlarged and rearranged when translated into Latin as *De augmentis scientiarum* in 1623; the former was addressed to the king and his entourage, the latter to professional philosophers.^58^ The significant differences in form and content between the two versions of the *Domestic and Field Pharmacy* is explained by this process of adapting texts for different audiences. It seems that the standard version of the *Domestic and Field Pharmacy* was designed for readers who had access only to standard medicines, not exotic or expensive ingredients. Gurchin and Blumentrost adapted their works to the practical needs of their servitor audience, in particular making the recipes relatively cheap and easy to create.

Gurchin and Blumentrost conceived their servitor audience rather differently from their patron audience. First and foremost, length, which appears to have been prized for a patron audience, here is stripped down to the minimum. Justifications for why a layperson would want to own a medical text are entirely practical: whereas such justifications in the texts intended for patrons cite appropriate knowledge and religious approval, in the texts intended for servitors it is simply noted that the text will be[Other P-722] useful. Just as with the patron audience, with the servitor audience the compilers had to justify their craft, but only in terms of its practical applications. The role of this text in changing the limits of literate medicine is also similar to that of the texts intended for patrons: they aimed to reduce the distance to literate medicine for a group who already had access to it.

## Commercial Literacy: Texts for Domestic Use

One group of texts created by Western European compilers is aimed at an audience entirely removed from official literate networks: households and ordinary laypersons. Such household texts, although a common genre elsewhere in Europe, were rare in Russia; the sole exemplar in Muscovy was the *Domostroi*. This work, which may have been partly taken from a Western European text, exists in multiple manuscripts from the mid-sixteenth century on.^59^ It deals with a range of issues pertinent to an urban household of moderate means: the disciplining of children, wives and servants; worship; arrangement and use of kitchen and garden; and various recipes. Among this miscellaneous advice there are statements on health and illness, which promote the view that illness is sent by God, and to try to heal oneself with medicines is wrong; one must instead pray for forgiveness and lead a good Christian life. It does mention folk healers, but these it condemns as sorcerers and forbids the reader from consulting them.^60^ The main household advice text available to seventeenth-century Russians thus counseled them to stay away from medicines and medical practitioners altogether.

Russian-language medical books of the sixteenth and early seventeenth centuries were rarely aimed at a lay, household readership. One of the most common Russian-language medical texts in the seventeenth century was the *Garden of Health* mentioned above.^61^ A herbal, it lists plants and enumerates their physical attributes and qualities; such a text might have been held by a private collector and could have been helpful for domestic medical practice, but it was not specifically designed to be used in the home. By the early eighteenth century, Russia had few household texts, and few medical texts aimed at a household audience.[Other P-723]

Given the lack of domestic medical texts, the fact that Apothecary Chancery men produced such texts is particularly interesting. In this case Gurchin and Blumentrost were not following an established Russian trend or tapping into an existing market, but creating a niche product. As with the other texts linked to the Apothecary Chancery, the works themselves reveal much about the intended audience. In common with the solders’ texts, these works state that they are to be used “in the absence of a doctor.” This phrase occurs in numerous eighteenth-century copies of the Russian *Pharmacopoeia*,^62^ as well as in the *Large Domestic Pharmacy* (1705).^63^ The phrase is significant, as the text presents itself not as replacing professional medical advice, but only as supplementing it in times of need.

The idea that a domestic medical text was only to be used in the absence of a trained medical professional is unusual. Typically, domestic medicine is seen as a gentle form of healing, as a first port of call and a prelude to calling in a professional if the ailment proved to be serious. Alternatively, the recent work of Seth Stein LeJacq has shown that at least some eighteenth-century English domestic recipe collections presented themselves as “strong” medicine, able to cope with serious illness instead of a physician or surgeon, or even to be used if the professionals failed.^64^ Although superficially these two purposes—before or after professional help—are starkly opposed, both presume that access to a medical professional would not be an issue. In contrast, Russian domestic recipe collections directly state that consulting a professional may be problematic.

This statement reveals an idea about the state of medical practice in Russia: that the audience of this book, middle-income townspeople, would commonly find themselves unable to consult a medical practitioner. It should be noted here that these texts, as being written by apothecaries and physicians, likely had a rather limited idea of what an appropriate professional was: a Western-trained practitioner like themselves, not an “old woman” such as the *Brief Description of Thirty Rules for Health* decried. It would thus seem that the medical provisions of the 1700s, even in urban centers, were thought by practitioners themselves to be so insufficient as to commonly fail to provide a literate practitioner when needed. Thus,[Other P-724] as with the works aimed at army servitors, Blumentrost and Gurchin designed their domestic works to fill a gap in the provision of literate medical experts.

The domestic texts also echo the servitors’ works in other ways. The *Large Domestic Pharmacy* states that its contents were “[c]ollected from many medical works,” echoing similar statements in the servitors’ texts.^65^ The works aimed at patrons also display this tendency to collect together material for a work: the *Reestr* is described by its title as being “of doctors’ knowledge,” potentially indicating that it gathered together information from various sources.^66^ The royal *Domestic and Field Pharmacy* presents many separate texts in one collection, also displaying this tendency of Gurchin’s texts to make a digest of material from various sources. That a medical work would collect together information from various sources is not unexpected, and in fact rather common for the period. The fact that this was so clearly stated in the preface is significant, however. By highlighting this fact, Gurchin and his collaborators assumed that creating a digest of information would be of greater interest than providing the whole text. Such an attitude is evident elsewhere in contemporary Russian medical affairs: Apothecary Chancery reports were based on Western European ideas and texts, but provided a short summary of the relevant information, rather than reproducing texts in their entirety. Gurchin and Blumentrost, when they created medical texts for laypersons, as with their texts for other groups, seem to have emulated the summarizing style they knew Russians to be interested in from their work at court.

In other ways the domestic works differ from the other lay medical texts compiled by Gurchin and Blumentrost. In contrast to the servitors’ works, which specify one particular group as the intended audience, or the patrons’ works, which specify one individual as the recipient, both the popular *Pharmacopoeia* and the *Large Domestic Pharmacy* state that “every person” will be able to use this text to heal themselves.^67^ The use of this phrase parallels English self-help medical texts of the same period, which frequently state that they were “for [use by] the meanest capacity,” meaning the poorest and least educated groups in society. The literacy rate in England was far above that of Russia, but even so people from the poorest level of society were illiterate, and so would not have been able to read such texts. This apparent disjunction between the text and data on literacy[Other P-725] rates has led some scholars to be extremely skeptical about the meaning of such prefaces, with Paul Slack describing them as “pious hopes or calculated advertisements rather than statements of fact.”^68^ Mary Fissell has similarly concluded that the hyperbolic statement about the suggested readership of these texts was a form of advertisement. Unlike Slack, Fissell does find a meaning in these prefaces, seeing them as indicating to potential buyers that they need not be versed in medical matters to use the text.^69^ Such an aim seems likely also to be true for the popular *Pharmacopoeia* and the *Large Domestic Pharmacy*. Here, Blumentrost and Gurchin choose to advertise their medicine not through a justification of its acceptability as a Christian activity, nor even as of practical utility, but rather by emphasizing that these texts were accessible to all who could read.

The phrase “every person” also leads us back to consideration of the limits of literate society in Russia. Inability to read a text does not mean inability to gain knowledge of its contents: as noted above, reports were commonly delivered to the tsar and his council orally; similarly, various witchcraft trials reveal that illiterate Muscovites relied upon the literate (or apparently literate) to read or use texts on their behalf.^70^ Writing of a much earlier period, Simon Franklin argues that Kievan church books reached a much wider audience than simply those who read them, by the very nature of their usage.^71^ One literate person in a friendship or kinship network could thus provide access to literate medical knowledge for a number of “illiterate” Muscovites. This could be the case for any of the texts under consideration here, but given the nature of this specific group of texts as “domestic” works aimed at “every person,” they seem particularly likely to have been a part of the nonliterate consumption of literate medicine through literate proxies.

These works also sometimes specify from where to purchase the drugs they suggest: the popular *Pharmacopoeia* states that it contains “a list of all medicines which are found in pharmacies [here meaning apothecary shops].”^72^ The preface focuses on medicines as an essential part of self-healing,[Other P-726] strongly suggesting that one should keep certain essential medicines at home. As made clear in the rest of the text, the word “medicines” does not mean only the herbs or roots that could be acquired from any source, but rather, there is an emphasis on minerals, chemicals, and other premixed, pharmaceutically prepared medicines that can be acquired, as stated in the preface, in apothecary shops. Before 1701 the only apothecary shop in Moscow was the Apothecary Chancery’s shop, opened in 1673; this was the only place that officially stocked pharmaceutical preparations.^73^ Moscow (and other towns) also had various market stalls selling herbal medicines, which did stock some such pharmaceutical medicines, but, following a series of deaths linked to those stalls, this market trade was outlawed as dangerous in 1701.^74^ Evidently, these “popular” editions of the *Pharmacopoeia* aimed to boost the Apothecary Chancery’s business by encouraging readers to purchase the pharmaceutical medicines they stocked.

The *Pharmacopoeia* goes on to mention that such pharmaceutical preparations need not be purchased only when necessary, but can be stored in medicine chests ready for usage, a practice the text describes as being common among “great persons.” This assertion was grounded in fact: a chest of medicaments was commonly sent with the tsar when he traveled outside Moscow.^75^ This practice was also apparently taken up by at least some boyars, as several boyars’ list of possessions, commonly compiled after death, included such a casket of medicines (*aptechka s lekarstvami*).^76^ Evidently, it was a practice the Apothecary Chancery wished to encourage. As with the reference to pharmaceutical medicines, the mention of chests of medicines was designed to increase sales in apothecary shops, once again demonstrating the role these texts played in promoting private medical and apothecary practice.

Works for laypersons thus assumed their audience to be practical-minded. Unlike the extensive justifications of medicine seen in patrons’ texts, and like the servitors’ texts, these domestic works simply state that they are useful. They openly acknowledge that they are not original, and indeed make a virtue of this fact, advertising themselves as digests of helpful knowledge. Such digests, the texts suggest, were particularly important given the low numbers of (appropriate) medical practitioners available: these works were explicitly designed to fill that gap. Western[Other P-727] medical practitioners here conceived their potential Russian patients and consumers as acting out of practical motives. These texts particularly push the boundaries of literate medicine: there is no indication that they were aimed at courtiers or others who had access to literate medicine. Rather, they sought to promote literate medicine to Russian literate society as a whole, notably expanding the sphere of literate medicine from exclusively a privilege of the court and the army.

## When Is a Medical Text Not a Medical Text? Satirical and Religious Appropriation of Medical Genres

The limits of literate medicine can be measured not only through medical texts themselves, but also through the existence of works that reference, emulate, and even deride medical works. Both appropriation and mockery require familiarity with the type of works being referenced, and so such works can be read against their explicit purpose of deriding medicine, to reveal how widespread knowledge of medical texts was essential to their very existence. Across the seventeenth and eighteenth centuries various so-called satirical *leechbooks* appeared in Russia,^77^ texts that used the format and language of medical recipe books to mock the content and worth of their serious counterparts (*leechbook* being a term for a kind of medical recipe book).^78^ The satirical leechbooks have commonly been viewed as a part of Russian xenophobia in their overtly negative attitude toward Western Europeans and Western European medicine. However, the texts themselves reveal a more complex relationship to Western European literate medicine.

The most famous such text, the *Leechbook for Foreigners*, survives in several eighteenth-century manuscripts, but was most likely first produced some time in the early seventeenth century. It begins with a preface that states that this book is “given by Russian people, how to heal foreigners and people of their land.”^79^ This work is openly hostile to foreigners, presenting the correct “healing” of foreigners as leading to their deaths.^80^ Other satirical works take healers as a target, with the seventeenth-century tale *Service to the Tavern* conflating healers (*lekary*) with confidence tricksters (*obmanshchiki*).^81^ Some actual hostility and violence toward foreign medical[Other P-728] practitioners did occur: in 1682 two Apothecary Chancery employees, Daniel von Gaden and Johann Guttbier, were killed by a mob, apparently due to suspicions that they had murdered Tsar Fedor Alekseevich. Yet this case does not display simple xenophobia: the mob targeted these men in particular, hunting von Gaden down over the course of several days, and did not attack Moscow’s foreign settlement as a whole.^82^ This reflects a wider conclusion of recent works about xenophobia in Muscovy: the number of polemical attacks seems to have outweighed actual attacks.^83^

The satirical leechbooks also attack medical knowledge and medical books. Indeed, it is medical recipes that these works most directly satirize. The *Leechbook for Foreigners* uses the same measuring terminology as other Russian medical recipes: *zolotnik* (4.26 grams) to measure dry goods, *kapel*’ (drop) to measure liquids. It also follows its serious counterparts in presenting medicines as complex, and in requiring the patient to take their medicines over a period of several days, often in combination with a specified program of rest, sleep, and diet. Ingredients in these recipes are either impossible (women’s folk dancing, chopped water) or impractical (sixteen *zolotiki* of a white bridge), or require the “patient” to undertake implausible activities, such as sweat in ice. Such deliberately bizarre content implies that the contents of serious medical recipes were similarly nonsensical.

The *Leechbook for Foreigners* was copied across the seventeenth century and into the eighteenth century, a period in which the role of foreigners in Russian medicine was expanding, and the numbers of foreign medical texts translated into Russian was growing. The 1534 *Garden of Health* was not only taken from a Western European work, it was translated into Russian by a German, Nicolaus Bülow; Gurchin and Blumentrost’s works of the late seventeenth and early eighteenth centuries continued the trend for foreigners providing Russians with medical texts.^84^ The *Leech-book for Foreigners* is an inversion of this situation: recipes by Russians for foreigners, rather than recipes by foreigners for Russians. The continued trend toward foreigners producing medical books for Russians might help explain the continued popularity of the *Leechbook for Foreigners*, as it remained relevant, satirizing the foreign medicine of the early eighteenth century as it had the foreign medicine of the early seventeenth century.[Other P-729]

Alongside such satirical texts, Russians also produced religious texts that appropriated medical formats, arranging religious aphorisms in the form of recipes for the improvement of spiritual health.^85^ These texts were by no means a marginal phenomenon: Tsar Fedor Alekseevich (r. 1676–82) owned one such text, called *On spiritual medicine* (*O dushestvennom lekarstve*).^86^ Such works for private devotion only became available in Russia in the latter part of the seventeenth century and the early eighteenth century as a part of the general expansion of Russian literate culture; previously, religious works had been for use in church and by priests and monks, not the laity.^87^ It is thus significant that this entirely new phenomenon of late seventeenth-century Russia—works for private devotion—would be produced in a manner that copied a medical format. Like the satirical leechbooks, these religious texts imitate the format of a medical recipe, by dictating how healing of the soul will only take place when certain “ingredients” are taken in a set manner. In the case of the religious texts, such “ingredients” are abstract concepts made flesh: “leaves of great patience,” “root of spiritual reconciliation.”^88^ As with the satirical texts, it is these nonexistent ingredients that most clearly distinguish these spiritual texts from serious medical works; otherwise, the format is notably similar.

This remarkable similarity of both the satirical leechbooks and these religious recipes with serious medical books indicates a distinct familiarity of their authors with those exact Western European texts. Such emulation of a format implies significant currency to the format within Russia: even those uncomfortable with Western medical texts acknowledged their importance. Moreover, both the satirical and the religious texts tell us something of their audience. In no satirical or religious text is there an explanation of the texts they emulate and deride. The authors thus assume that any reader of their text would be fairly familiar with the very medical texts against which the author is arguing. This is most evident in the case of the devotional works: these texts were themselves a new phenomenon for late seventeenth-century Russia, but some of the earliest examples use the medical format, implying that by that time literate Russians were likely to have been fairly familiar with the medical works they emulate. The satirical and religious texts discussed here do not seek to redraw the limits of literate medicine, but instead assume those limits[Other P-730] to lie far beyond official court medicine, in the general realm of Russian literate culture.

## Conclusion

First and foremost, what the works surveyed above show is that their compilers believed that the limits of literate medicine in Russia lay not at the edges of the court, but at the edges of literate society. Although some of these works were for specific patrons—Tsar Peter the Great, Tsarevich Aleksei, Military Governor Apraksin—many others were not designed with such a specific audience in mind, and seem to have been intended to be sold to any who would buy. Medical texts were promoted by Gurchin and Blumentrost not as specialist reading material for practitioners alone, but rather as part of a general readers’ library. The idea that medical works had a place in literate Russian culture is supported by the so-called satirical medical books, and the religious books that similarly copied medical genres. Despite their obvious aim of denigrating serious medical works, these texts in fact underline the place that medicine had in literate culture, by demonstrating the familiarity of their authors with such serious medical texts, and assuming such familiarity in their audience.

Familiarity with medical texts did not automatically lead to acceptance of Western European medicine, or preference for Western European literate medicine over native nonliterate healing. Gurchin and Blumentrost take pains, particularly in their earlier works for patrons, to justify medicine as appropriate knowledge. In texts from the 1690s, multiple such strategies are employed, presenting medicine in particular as godly and worthy of royal attention. In the 1700s and 1710s, and in the more general works, the only defense made of medicine is that it is useful. Nevertheless, in all works there is some attempt to justify the idea of a medical text for a layperson, showing that Gurchin and Blumentrost did not feel that their Russian audience was entirely won over to Western European medicine. These medical texts thus served to further advance the cause of Western European practitioners of literate medicine in Russia.

Indeed, these texts seem to have occupied a key place in the strategy of men such as Gurchin and Blumentrost. Having worked productively for the court, they also wished to make a place for themselves in the wider Russian medical world. As several of these texts explicitly acknowledge, medical provision, at least of Western European–style practitioners, was severely limited. Such limitations did not only apply to servitors, but also to townspeople. Thus the importance of the Western European–style texts was in continuing the provision of Western European medicine even[Other P-731] without Western European practitioners, and so discouraging recourse to native Russian practitioners and practices. Despite the low level of literacy in early eighteenth-century Russia, texts for laypersons nevertheless played a role in shaping and reshaping the sphere of literate medicine in Russia.[Other P-732]

## Footnotes

1
LevinEveHealers and Witches in Early Modern RussiaSaluting Aron Gurevich: Essays in History, Literature, and Other Related SubjectsMazour-MatusevichYelenaKorrosAlexandra S.LeidenBrill2010105133

2
FranklinSimonBooklearning and Bookmen in Kievan Rus’: A Survey of an IdeaHarvard Ukrainian Stud12/131988/89830848

3
KivelsonValerieWhat Was *Chernoknizhestvo*? Black Books and Foreign Writings in Muscovite MagicRude and Barbarous Kingdom RevisitedDunningChesterMartinRussellRowlandDanielBloomington, Ind.Slavica2008115 KivelsonDesperate Magic: The Moral Economy of Witchcraft in Seventeenth-Century RussiaIthaca, N.Y.Cornell University Press2013133151

4
MaierIngridPilgerWouterSecond-Hand Translation for Tsar Aleksej Mixajlovic—A Glimpse into the ‘Newspaper Workshop’ at *Posol’skij Prikaz*Russian Linguistics252001209242

5
MarkerGaryLiteracy and Literacy Texts in Muscovy: A ReconsiderationSlavic Rev4919907489 WitzenrathChristophLiteracy and Orality in the Eurasian Frontier: Imperial Culture and Space in Seventeenth-Century Siberia and RussiaSlavonic East European Rev8720095377 StevensCarol B.Belgorod: Notes on Literacy and Language in the Seventeenth-Century Russian ArmyRussian Hist71980113124

6
BushkovitchPaulPeter the Great: The Struggle for Power, 1671–1725CambridgeCambridge University Press2001303

7
GrombakhS. M.Russkaia meditsinskaia literatura XVIII vekaMoscowAkademia meditsinskikh nauk1953 RennerAndreasRussische Autokratie und europäische Medizin. Organisierter Wissenstransfer im 18 JahrhundertStuttgartFranz Steiner2010 FranklinSimonPrinting and Social Control in Russia 1: PassportsRussian Hist372010208237 FranklinPrinting and Social Control in Russia 2: DecreesRussian Hist382011467492

8
GruzdevV. F.Russkie rukopisnye travnikiLeningradVoenno-morskoi meditsinskaia akademiia1946517 IppolitovaA. B.Russkie rukopisnye travniki XVII-XVIII vekov. Issledovanie fol’klora i etnobotanikiMoscowIndrik20082349

9
RichterWilhelmGeschichte der Medicin in Russland3MoscowN. S. Vsevoloskii1813–17 ZagoskinN. P.Vrachi i vrachebnoe delo v starinnoi RossiiKazanTipografiia Imperatorskogo universiteta1891 NovombergskiiN. Ia.Cherty vrachebnoi praktike v Moskovskoi Rusi (kultur’no-istoricheskii ocherk)St. PetersburgTipografiia Ministerstva vnutrennykh del1904 NovombergskiiN. Ia.Nekotorye spornye voprosy po istorii vrachebnogo dela v do-Petrovskoi RusiSt. PetersburgTipografiia Ministerstva vnutrennykh del1903 MirskiiM. B.Meditsina Rossii X-XX vekov. Ocherki istoriiMoscowROSSPEN2005 MirskiiM. B.Ocherki istorii meditsiny v Rossii XVI-XVIII vvVladikavkaz1995 UnkovskayaMariaLearning Foreign Mysteries: Russian Pupils of the Aptekarskii Prikaz, 1650–1700Oxford Slavonic Pap301997120 UnkovskayaMariaBrief Lives: A Handbook of Medical Practitioners in Muscovy, 1620–1701LondonWellcome Trust1999 DumschatSabineAusländischer Mediziner im Moskauer RusslandStuttgartFranz Steiner2006 LevinEveThe Administration of Western Medicine in Seventeenth-Century RussiaModernizing Muscovy: Reform and Social Change in Seventeenth Century RussiaKotilaineJarmoPoeMarshallLondonRout-ledge Curzon2004353379 SokolovskiiM.Kharakter i znachenie deiatel’nosti Aptekarskogo prikazaSt. PetersburgP. P. Soikin1904

10 See Unkovskaya, “Learning Foreign Mysteries” (n. 9).

11
AlexanderJohn T.Medical Developments in Petrine RussiaCanadian-American Slavic Stud81974198221

12
OreshnikovO.Danil Gurchin. Moskovskii aptekar’ nachala XVIII vekaSbornik statej v chest’ grafini Praskov’i Sergeevny UvarovojMoscow19165455

13
MorozovB. N.Travnik iz Postel’noi kazny Ivana Groznogo? Khar’kovskaia rukopis’ 1534g.—novyi pamiatnik knizhnoi masterskoi mitropolita Daniila (Pervye itogi izucheniia)Arkheograficheskii ezhegodnik za 2002 godMoscowNauka20047385

14
ZmeevL. F.Russkie vrachebniki. Issledovanie v oblasti nashei drevnei vrachebnoi pis’mennostiPamiatniki drevnei pis’mennosti i iskustva112St. Petersburg1896 UtkinL. A.GammermanA. F.NevskiiV. A.Bibliografiia po lekarstvennym pasteniiam. Ukazatel’ otechestvennoi literatury: rukopisi XVII-XIX vv., pechatnye izdaniia 1732–1954MoscowIzdatelstvo Akademii Nauk SSSR1957

15
Savel’evaE. A.Katalog knig iz sobraniia Aptekarskogo prikazaSt. PetersburgAl’faret2006 KhoteevP. I.Biblioteka Leib-medika I. G. LestokaKniga i biblioteki v Rossii v XIV—pervoi polovine XIX vLuppovS. P.LeningradBAN19824255

16
BogoiavlenskiiN. A.Drevnerusskoe vrachevanie v XI-XVII vv.. Istochniki dlia izucheniia istorii russkoi meditsiny727998 ZmeevRusskie vrachebniki101114264

17
OreshnikovDanil Gurchin5455

18
PanichT. V.Literaturnoe tvorchestvo Afanasiia KholmogorskogoNovosibirskSibirskii khronograf1996124125

19 Lit. “baggage train.”

20
GriffinClareThe Production and Consumption of Medical Knowledge in Seventeenth-Century Russia: The Apothecary ChanceryPh.D. diss.University College London2013

21
OreshnikovDanil Gurchin5658

22
UnkovskayaBrief Lives29 DumschatAusländische Mediziner569574

23
DumschatAusländische Mediziner569572

24
LeongElainePennellSaraRecipe Collections and the Currency of Medical Knowledge in the Early Modern ‘Medical Marketplace,’Medicine and the Market in England and Its Colonies, c. 1450–c.1850JennerMarkWallisPatrickBasingstokePalgrave Macmillan2007133152

25
WaughDaniel ClarkeThe Library of Aleksei MikhailovichForschungen zur osteuropäischen Geschichte381986299324

26
BrownPeter B.How Muscovy Governed: Seventeenth-Century Russian Central AdministrationRussian Hist362009459529

27
CrummeyRobert O.The Origins of the Noble Official: The Boyar Elite, 1613–1689Russian Officialdom: The Bureaucratization of Russian Society from the Seventeenth to the Twentieth CenturyRowneyD. K.PintnerW. M.Chapel HillUniversity of North Carolina Press19804675

28
MamonovN. E.Materialy dlia istorii medistiny v Rossii4St. PetersburgM. M. Stasiulevich18812157 MamonovN. E.Materialy dlia istorii medistiny v Rossii4St. PetersburgM. M. Stasiulevich188121573636639

29 RNB, kollektsiia A. A. Titova, no. 3881 (Royal *Domovaia i pokhodnaia apteka*, early eighteenth century), State Historical Museum [GIM] sobr. Uvarova, no. 172 (Royal *Domovaia i pokhodnaia apteka*, early eighteenth century).

30
PrussakObzor2425 SokolovskiiKharakter85

31
CracraftJamesThe Petrine Revolution in Russian CultureCambridge, Mass.Harvard University Press2004240241

32 RNB, koll. Titova, no. 3881 (n. 29).

33 “по б[o]жией воли подается питателство и богаство особно в ыныхъ г[o]с[у]д[a] рствахъ иземляет … сверхъ воздержитъ ч[e]л[o]в[e]ческое здравие,” RNB koll. Titova, no. 3881 (n. 29).

34 “покорно уничиженно воспаметовать изволтъ м[и]л[o]стиво разсуждати с библейныя притчи в Моисееве Книге … какъ Моисе несозженное злато в порохъ сожекъ и людем вадалъ пити в воде, и какъ Ц[a]рь Соломонъ сие познавание руд и всехъ трав и деиства ихъ в великой чести воздержалъ писано въ Книге Соломоновой,” RNB koll. Titova, no. 3881 (n. 29).

35
FlorinskiiV. M.Russkie prostonarodnye travniki i lechebniki. Sobranie meditsinskikh rukopisei XVI i XVII stoletiiaKazanTipografiia Imperatorskogo universiteta1879211229 PanichLiteraturnoe tvorchestvo191206 PrussakObzor2728 GruzdevRusskie rukopisnye3536 ZmeevRusskie vrachebniki133138 BogoiavlenskiiDrevnerusskoe vrachevanie101102 ViktorovA.Sobranie slaviano-russkikh rukopisej V. M. Undolskogo. Bibliograficheskii ocherkMoscowUniversitetskaia tipografiia187028

36
PanichLiteraturnoe tvorchestvo125126

37 “которые суть к человеческим немощам прилично держать лекарства и те лекарства к какой причине быти прилежат, и как к немощам человеческим составит водки и из каких зелий, так те ж лекарства из каких вещей и какую они имеют силу, ибо тому прилично быть у Вашего графскаго сиятелства и ближнаго столника Феодора Матвеевича Апраксина,” Panich, *Literaturnoe tvorchestvo* (n. 18), 129–30.

38
KivelsonDesperate Magic148149 HughesLindseyRussia in the Age of Peter the GreatNew Haven, Conn.Yale University Press1998251 RyanW. F.The Bathhouse at MidnightStroudSutton1999415416

39 Kivelson, “What Was *Chernoknizhestvo*?” (n. 3).

40
KivelsonDesperate Magic150

41 RNB koll. Titova, no. 3881 (n. 29), folios 17-30.

42 RNB koll. Titova, no. 3881 (n. 29), folios 31–32.

43 “непослушаите всякаго неискуснаго соседа или сродственика в совете в лекарствах,” RNB koll. Titova, no. 3881 (n. 29), folios 31–32.

44 “недаваите себя лечити молодымъ лекарямъ и старымъ бабамъ а будъ в вас болезнь или рана припадетъ посоветуите всегда в лекарстве искусно,” RNB koll. Titova, no. 3881(n. 29), folios 31–32.

45 See RGADA (Russian State Archive of Ancient Documents) f. 143, op. 2, ed. khr. 1251 and Mamonov, *Materialy* (n. 28), 4:1110–11 (1679 malpractice case against Grigorii Donskoi); N. Ia. Novombergskii, *Materialy po istorii meditsiny v Rossii*, 4 vols. (St. Petersburg: Tip. M. M. Stasiulevicha, 1905), 2:311–12 (1686 malpractice case against Andrei Kharitonov and Mikhail Tuleishchikov); RGADA f. 143, op. 3, ed. khr. 462 (1699 investigation of herbal medicine stalls).

46
DumschatAusländischer Mediziner202203

47
OreshnikovDanil Gurchin48

48
BogoiavlenskiiS. K.Prikaznye sud’i XVII vekaMoscowAkademii nauk19461316

49
ZitserErnest A.The Transfigured Kingdom: Sacred Parody and Charismatic Authority at the Court of Peter the GreatIthaca, N.Y.Cornell University Press200456186193

50 “которою егда лекаря нетъ могутъ сами себе помощи дать во всякихъ своих и конскихъ немощехъ,” RNB kollektsiia M. P. Pogodina, no. 1561 (collection including *Apteka obozovaia ili sluzhivaia*, eighteenth century), folio 110.

51
UnkovskayaLearning Foreign Mysteries1516

52
AlexanderMedical Development207

53 “Собранная вкратце,” RNB kollektsiia M. P. Pogodina, no. 1561 (n. 50), folio 110.

54 RNB koll. M. P. Pogodina, no. 1561 (n. 50).

55 “которою егда лекаря нетъ могутъ сами себе помощи дать во всякихъ своих и конскихъ немощехъ,” RNB kollektsiia M. P. Pogodina, no. 1561 (n. 50), folio 110.

56
SokolovskiiKharakter85 DumschatAusländischer Mediziner571 PrussakObzor2425

57 RNB koll. Titova, no. 3881 (n. 29), folios 4–4ob.

58
PantinIsabelleThe Role of Translations in European Scientific Exchanges in the Sixteenth and Seventeenth CenturiesCultural Translation in Early Modern EuropeBurkePeterHsiaR. Po-ChiaCambridgeCambridge University Press2007163179

59
The Domostroi: Rules for Russian Households in the Time of Ivan the TerriblePouncyCarolyn JohnstonIthaca, N.Y.Cornell University Press19944243

60
DomostroiKolesovV. V.RozhdestvenskaiaV. V.St. PetersburgNauka199415

61
IsachenkoT. A.Perevodnaia Moskovskaia knizhnost’. Mitropolichii i patriarshii skriptorii XV-XVII vvMoscowRossiiskaia gosudarstvennaia biblioteka2009135153

62 “в не бытий доктора,” GIM sobranie rukopisei I. E. Zabelina, no. 674 (*Pharmacopoeia*, eighteenth century), folio 1–ob. See also GIM sobr. Vakhrameeva, no. 534; GIM sobr. Barsova, no. 2238.

63 “егда лекаря нетъ,” RNB f. 550, VI, Quartos, no. 45, folios 86–162v. The 1779 manuscript Oreshnikov cites states the date of composition of the *Large Home Pharmacy* as 1708, not 1705. Oreshnikov, “Danil Gurchin” (n. 12), 54.

64
LeJacqSeth SteinThe Bounds of Domestic Healing: Medical Recipes, Storytelling and Surgery in Early Modern EnglandSoc. Hist. Med262013451468

65 RNB f. 550, VI, Quartos, no. 45, folios 86–162v.

66
PanichLiteraturnoe tvorchestvo124126130133

67 For the *Pharmacopoeia*, see GIM sobranie rukopisei I. E. Zabelina, no. 674 (*Pharmacopoeia*, eighteenth century), folio 1–ob; GIM sobr. Vakhrameeva, no. 534; GIM sobr. Barsova, no. 2238. For the *Large Domestic Pharmacy*, see RNB f. 550, VI, Quartos, no. 45, folios 86–162v.

68
SlackPaulMirrors of Health and Treasures of Poor Men: The Uses of the Vernacular Medical Literature of Tudor EnglandHealth, Medicine, and Mortality in the Sixteenth CenturyWebsterCharlesCambridgeCambridge University Press1979237273

69
JennerWallisMedicine and the Market108132

70
KivelsonDesperate Magic133151

71
FranklinSimonWriting, Society, and Culture in Early Rus, c. 950–1300CambridgeCambridge University Press200234

72 “преписание всехъ лекарствъ: которые обретаются во аптекахъ,” GIM sobranie rukopisei I. E. Zabelina, no. 674 (n. 62), folio 1–ob. See also GIM sobr. Vakhrameeva, no. 534; GIM sobr. Barsova, no. 2238 (n. 62).

73 Levin, “Administration” (n. 9), 357.

74 Oreshnikov, “Danil Gurchin” (n. 12), 48.

75
MamonovMaterialy2234

76
OrlenkoS. P.Vykhodtsy iz zapadnoi Evropy v Rossii XVII veka. Pravovoi status i real’noe polozhenieMoscowDrevnekhranilishche2004175

77 “Leechbook” here is a translation of the Russian *lechebnik*.

78
BoevaL. I.Lechebniki i retsepty kak formy satiricheskogo oblicheniiaDrevniaia Rus’. Voprosi medievistiki1420035662

79
Adrianova-PerettsV. P.Russkaia demokraticheskaia satira XVII vekaMoscowAkademii Nauk1954121

80
BoevaLechebniki59

81
BoevaLechebniki56

82
DumschatSabineKar’era pridvornogo vracha Daniila Fungadanova v svete sovremennykh kultur’no-istoricheskikh issledovanii ob inostrannykh medikakh v Moskovskom gosudarstve 15–17 vekovInozemtsy v Rossii v XV-XVII vekakh. Sbornik materialov konferentsii 2002–2004 ggLevykinA. K.MoscowDrevlekhranilishche2006356367

83
OparinaT. A.Inozemtsy v Rossii XVI-XVII vvMoscowProgress-Traditsiia2007

84
MillerDavidThe Lübeckers Bartholomäus Ghotan and Nicolaus Bülow in Novgorod and Moscow and the Problem of Early Western Influences on Russian CultureViator. Mediev. Renaiss. Stud91978395412

85
GruzdevRusskie rukopisnye2021

86
SedovP. V.Zakat Moskovskogo tsarstva. Tsarskii dvor kontsa XVII vekaSt. PetersburgDmitrii Bulanin2008179

87
HughesRussia344345

88
Adrianova-PerettsRusskaia demokraticheskaia satira282

